# The impact of steatotic liver disease on coronary artery disease through changes in the plasma lipidome

**DOI:** 10.1038/s41598-024-73406-8

**Published:** 2024-09-27

**Authors:** Elias Björnson, Dimitrios Samaras, Malin Levin, Fredrik Bäckhed, Göran Bergström, Anders Gummesson

**Affiliations:** 1 Wallenberg Laboratory, Department of Molecular and Clinical Medicine, Sahlgrenska Center for Cardiovascular and Metabolic Research, University of Gothenburg, Gothenburg, 413 45 Sweden; 2 Region Västra Götaland, Department of Clinical Physiology, Sahlgrenska University Hospital, Gothenburg, 41345 Sweden; 3 Region Västra Götaland, Department of Clinical Genetics, Sahlgrenska University Hospital, Gothenburg, 413 45 Sweden

**Keywords:** Mediation analysis, Fatty acid, Lipidomics, Sphingolipids, Glycerophospholipids, LDL, Triglycerides, Atherosclerosis, Cardiology, Medical research, Molecular medicine

## Abstract

Steatotic liver disease has been shown to associate with cardiovascular disease independently of other risk factors. Lipoproteins have been shown to mediate some of this relationship but there remains unexplained variance. Here we investigate the plasma lipidomic changes associated with liver steatosis and the mediating effect of these lipids on coronary artery disease (CAD). In a population of 2579 Swedish participants of ages 50 to 65 years, lipids were measured by mass spectrometry, liver fat was measured using computed tomography (CT), and CAD status was defined as the presence of coronary artery calcification (CAC score > 0). Lipids associated with liver steatosis and CAD were identified and their mediating effects between the two conditions were investigated. Out of 458 lipids, 284 were found to associate with liver steatosis and 19 of them were found to also associate with CAD. Two fatty acids, docosatrienoate (22:3n6) and 2-hydroxyarachidate, presented the highest mediating effect between steatotic liver disease and CAD. Other mediators were also identified among sphingolipids and glycerophospholipids, although their mediating effects were attenuated when adjusting for circulating lipoproteins. Further research should investigate the role of docosatrienoate (22:3n6) and 2-hydroxyarachidate as mediators between steatotic liver disease and CAD alongside known risk factors.

**Supplementary Information:**

The online version contains supplementary material available at 10.1038/s41598-024-73406-8.

## Introduction

Steatotic liver disease is recognized as the most prevalent liver disease, affecting 17–51% of adults worldwide^1^. It is commonly associated with the metabolic syndrome, which is characterized by obesity, insulin resistance, diabetes, hypertension, and dyslipidemia. Several studies, based on imaging-defined atherosclerosis as well as cardiovascular events, have reported that individuals with liver steatosis have a significantly increased risk of developing cardiovascular disease^2–5^. We have shown that the association between liver steatosis and coronary artery calcification score (CACS), which is a common measurement for the estimation of coronary artery disease (CAD), remains strong even after adjusting for other metabolic syndrome factors^6^. Despite this established association, there is a lack of knowledge regarding the mechanisms connecting these conditions.

Dyslipidemia is a significant risk factor for CAD and has been consistently associated with liver steatosis, even after adjusting for other metabolic risk factors which underscores the profound influence of the liver’s condition on lipid levels^7^. Changes in lipid metabolism induced by liver steatosis have been demonstrated to promote atherosclerosis through lipoproteins^8^. More specifically, triglyceride-rich lipoproteins have been found to mediate approximately 15% of the association between liver steatosis and CACS, while LDL cholesterol to mediate about 5%^9^. However, these lipoproteins do not encompass the entire impact of liver steatosis on circulating lipids, and a significant portion of the association between liver steatosis and CAD remains to be elucidated. Hence, the aim of this study is to identify potential lipids linking steatotic liver disease to CAD using plasma lipidomics.

## Methods

### Study population

This investigation includes two populations: the Impaired Glucose Tolerance and Microbiota Study (IGT-Microbiota study) and a sub-population of the Swedish CArdioPulmonary bioImage Study (SCAPIS). IGT-Microbiota is an observational study of randomly selected Swedish individuals aged 50 to 65, with 1965 participants selected from over 5191 screened individuals, categorized by glycemic status so that they reflect a wide range of glucose levels^10^.Participants were recruited if they had dysglycemia (diabetes, impaired fasting glucose and/or impaired glucose tolerance) based on fasting glucose values and an oral glucose tolerance test (OGTT). Individuals were also included if they showed increased risk for future diabetes according to the Finnish Diabetes Risk Score (FINDRISC, score > 14) or had 2 first-degree relatives with diabetes. Individuals with normal glucose tolerance and FINDRISC score ≤ 14 were randomized (1:4) to inclusion. Exclusion criteria were diabetes, serious illness such as inflammatory bowel disease, rheumatic diseases, treatment with steroids, immune-modulating drug use, malignancy, antibiotic use within the last 3 months, and major cognitive dysfunction. SCAPIS is an observational study of 30,154 people aged 50 to 65 examined at six different sites in Sweden. Only the inability to comprehend spoken and written Swedish required for informed consent constituted an exclusion criterion in SCAPIS. The current study involves a subcohort consisting of 1111 participants who were selected from the SCAPIS Gothenburg site to match the traits of the IGT-Microbiota study population^11^ by applying the same inclusion/exclusion criteria as for the IGT-Microbiota study and selection based on glucose measurements to obtain similar proportions of individuals with dysglycemia and normal glucose tolerance. Cross-sectional data at baseline from these two populations were combined, resulting in a total study population of 2579 after excluding individuals on lipid-lowering therapy or with missing CACS. All participants provided written informed consent. The IGT-Microbiota study was approved by the ethics committee at Gothenburg University (Dnr 560 − 13), and SCAPIS was approved as a multicenter study by the ethics committee at Umeå University (Dnr 2010-228-31 M). The use of their data for analysis was approved by the Swedish ethical review authority in Uppsala (Dnr 2021–04030). All procedures performed were in accordance with the ethical standards of the Declaration of Helsinki.

### Measurements

#### Liver steatosis measurements

Computer tomography (CT) was used for estimating liver steatosis. Subjects fasted at least 4 h before the CT visit. To standardize the liver glycogen levels the participants were given a standardized meal (Modifast, Nutriton&Santé) calculated based on body mass index (BMI) two hours prior to CT examination^12^. A dedicated dual-source CT scanner equipped with a Stellar Detector (Somatom Definition Flash, Siemens Medical Solution, Forchheim, Germany) was used for measuring CACS and fat deposits^11^. Liver fat estimation in CT scans is based on lower brightness due to fat’s reduced radiation absorption compared to liver tissue, quantified as mean attenuation in Hounsfield units (HU). A single CT scan slice with thickness of 5 mm in the level of the fourth lumbar vertebra (L4) depicting both liver lobes and the spleen was used to estimate a mean liver attenuation value^12^. Algorithms were used for automated image analysis, providing a range from 2 to 74 HU in the population of this study. Liver attenuation was used as a continuous variable in our study with its values subtracted from 100 to have a value in positive relation to the degree of hepatic steatosis and then logged transformed to achieve normal distribution. This variable was used as an indicator of liver steatosis.

#### Coronary artery calcium score measurements

Calcium content in the coronary arteries was measured using CT as previously described^11^. In brief, electrocardiogram-gated noncontrast CT imaging at 120 kV was used to get calcium content images. These images were reconstructed by using B35f HeartView medium CaScore, while CACS was estimated by the use of syngo.via calcium scoring software (Volume Wizard; Siemens)^13^. The amount of calcified content found in coronary arteries was summed to create the total CACS according to international standards^14^. CACS was used as a binary variable, categorizing participants into two groups: those with a CACS of zero and those with a CACS > 0 indicating atherosclerotic plaque presence.

#### Clinical covariates

Data on age, sex, and alcohol consumption were collected using standardized questionnaires. Alcohol consumption (ordinal-scale variable) was based on frequency using the question “How often do you have a drink containing alcohol?”, corresponding to the first question in the Alcohol Use Disorders Identification Test (AUDIT). Body weight was measured in light clothing without shoes. Systolic and diastolic blood pressure were measured in the supine position with an automatic device (Omron M10-IT, Omron Health care Co, Kyoto, Japan). Clinical chemistry analyses included HDL-C, TG, LDL-C, plasma glucose, hemoglobin A1c (HbA1c), insulin, and C-reactive protein (CRP)^11^.

### Lipidomics analysis

Venous blood samples were collected after an overnight fast of at least 8 h and were centrifuged and stored at − 80 °C. Maximum time from needle-to-freeze was 2 h. Plasma samples from the participants were analyzed by Metabolon, Inc (Durham, NC) using Ultrahigh Performance Liquid Chromatography-Tandem Mass Spectroscopy (UPLC-MS/MS)^15^. Samples were prepared using the automated MicroLab STAR^®^ system from Hamilton Company where proteins were removed. Raw data was extracted, peak-identified and quality control processed using Metabolon’s hardware and software. The retrieved values were normalized by dividing each metabolite by its median value across all samples in each batch. The imputation process conducted by Metabolon was to replace missing values with the observed minimum value for each metabolite after batch normalization when the missing values were not due to random processing error. The total number of metabolites that were assigned to the lipids category by Metabolon, Inc in the dataset were 458 and were thus included in this study.

### Statistical methods

#### Lipids’ association with liver steatosis and CACS

Associations between plasma lipids and liver steatosis were determined in linear regression models adjusted for age, sex, alcohol consumption, and cohort (IGT-Microbiota study or SCAPIS). To test for associations between plasma lipids and CACS = 0 compared to CACS > 0, we used logistic regression models adjusted for age, sex, and cohort. P-values were adjusted for multiple comparisons using the false discovery rate method and the lipids significantly associated with both liver steatosis and CACS group (p-value < 0.05) constituted the dataset used in the mediation analysis.

#### Mediation analysis

Mediation analysis assesses relationships between variables using mediators. In this study, we used the package *mediation* in R. Briefly, the analysis involves two regression models: one for the independent variable’s effect on mediators and one for mediators’ effects on the dependent variable^16^. The covariates used in the mediation model were age, sex, study cohort and alcohol consumption and liver attenuation was logged and coded as a continuous variable, as opposed to using a cut-off value. The output of this process includes the value for the average causal mediation effect (ACME), namely the effect of the independent variable (i.e., liver fat) on the dependent variable (i.e., CACs) moving indirectly through the mediator (i.e., the plasma lipid), as well as the value for the average proportion mediated. Confidence intervals for the ACME values were obtained using the bootstrapping method provided by the mediation package. Sensitivity analysis was performed to examine how various metabolic risk factors affected the lipid-mediated effects by including body mass index (BMI), Homeostatic Model Assessment for Insulin Resistance (HOMA-IR), systolic blood pressure (SBP), C-reactive protein (CRP), LDL-cholesterol (LDL-C), and plasma triglycerides (TG), one at a time in the mediation model.

## Results

### 284 lipids associated with steatotic liver disease, 19 of them also with CACS > 0

**Fig. 1:**
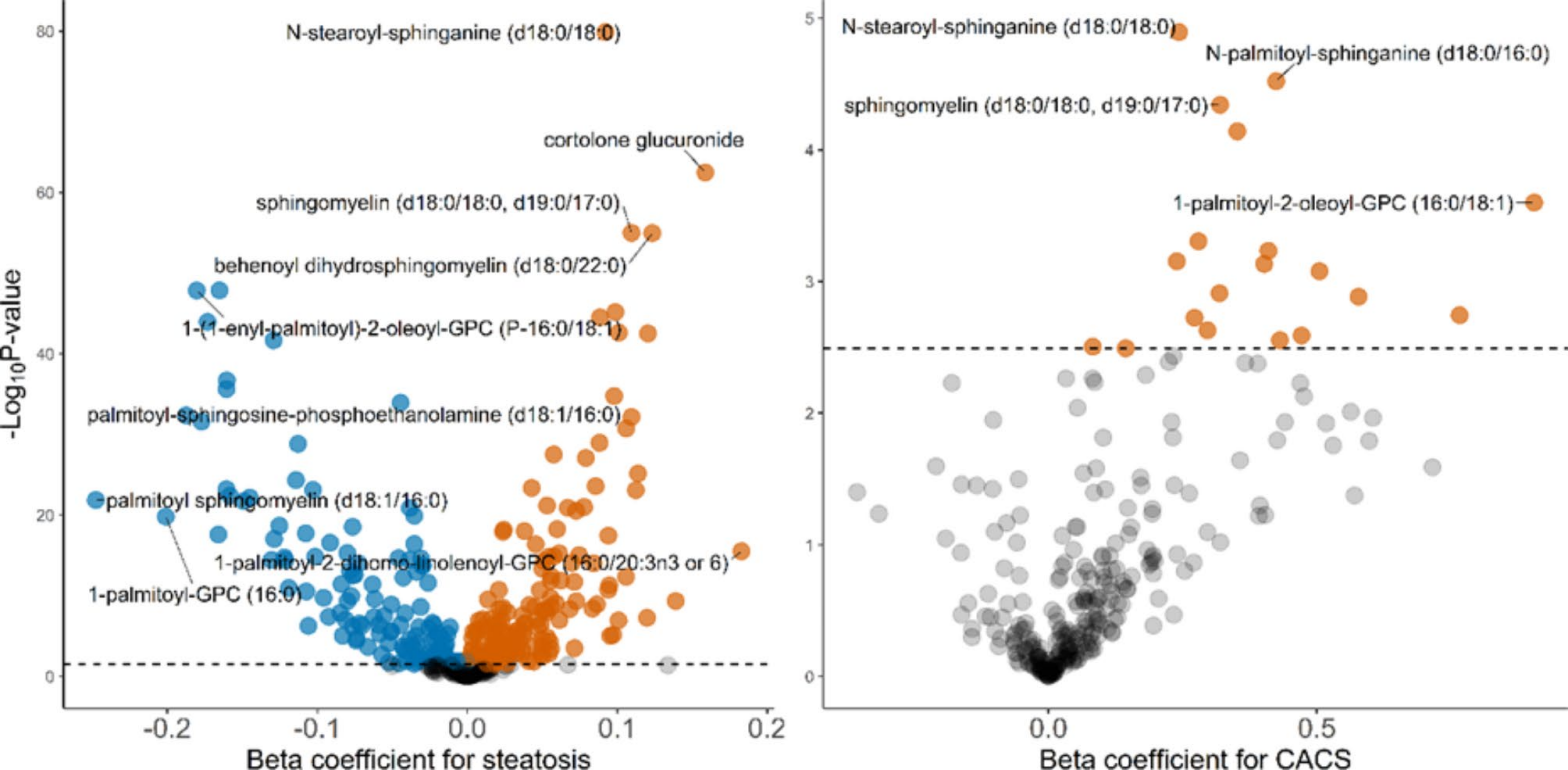
Volcano plots of regression models of liver steatosis and CACS on lipids. The x-axis represents the beta coefficient of each lipid in the corresponding regression model, while the y-axis shows the negative logarithm of the respective adjusted p-values.

**Fig. 2:**
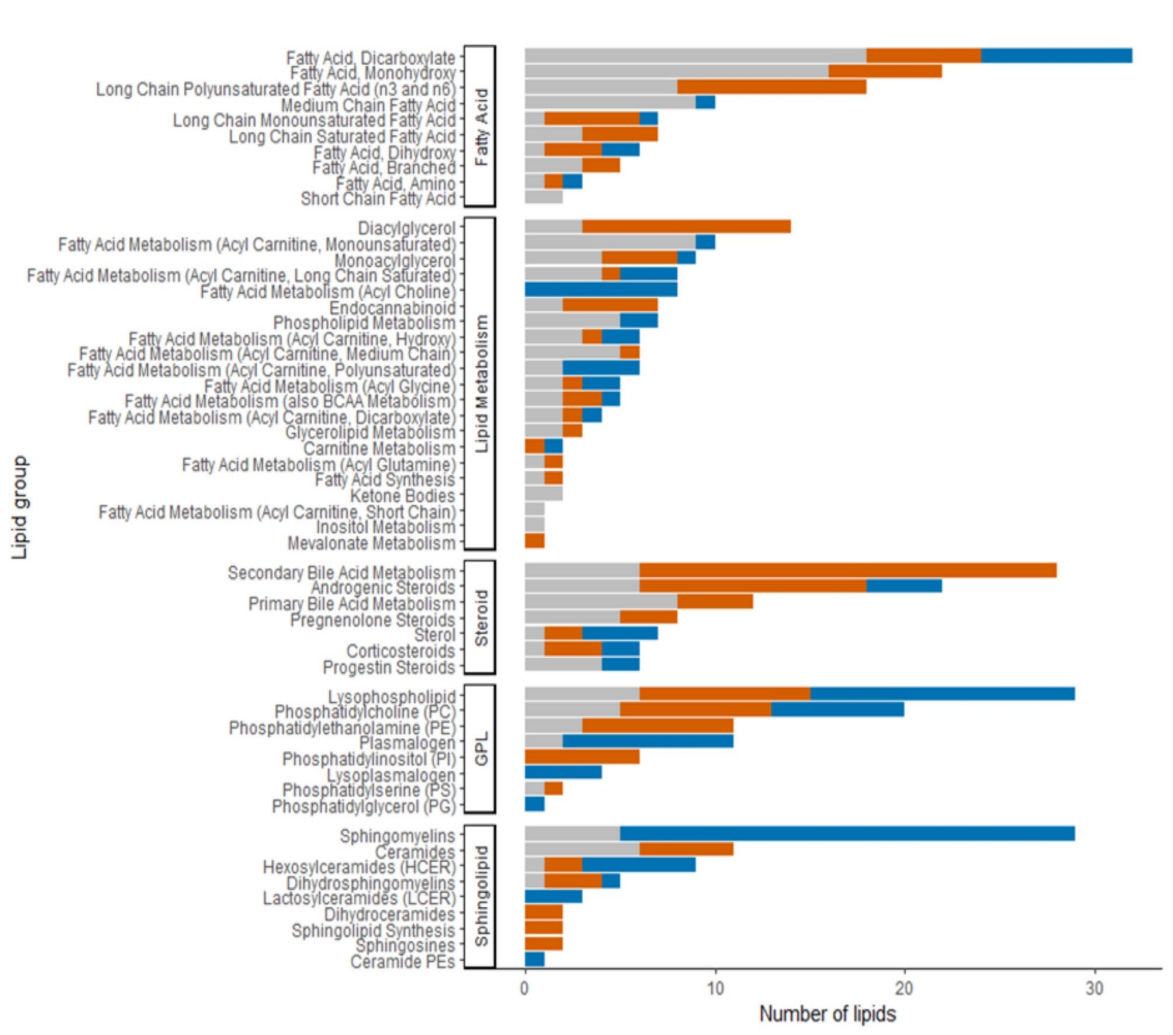
Liver steatosis’ lipidomic profiling with groups and subgroups of lipids ordered by number of lipids. Bar chart presenting how many lipids in each group and subgroup were found to be unassociated (grey), increased (red), or decreased (blue) in liver steatosis.

Of the 2579 individuals of Swedish descent, 46,6% were male and the mean age was approximately 58 years. Half of the population had liver attenuation > 57 HU denoting no liver steatosis, while 1426 individuals had a CACS of zero and 1153 individuals had CACS > 0 (Table 1). Out of 458 lipids, 284 (62%) were significantly associated with liver steatosis (Fig. 1). We also categorized the lipids into groups and subgroups to give an overall view of the plasma lipidomic changes in steatotic liver disease (Fig. 2). The model included adjustments for age, gender, cohort, and alcohol consumption, while the additional inclusion of BMI did not alter the overall profile (**Supplementary Fig. 1**).

**Table 1:** Descriptive statistics for the IGT-study and SCAPIS, as well as their combined populations used in this study.

	IGT-Microbiota	SCAPIS		Total population	
n	1679	900		2579	
Age	57.86 (4.52)	58.22 (4.26)		57.99 (4.44)	
Male (n, %)	752 (44.8)	451 (50.1)		1203 (46.6)	
BMI, kg/m^2^	27.59 (4.31)	27.43 (4.85)		27.53 (4.50)	
*Degree of steatosis (n, %)*					
No Steatosis (HU > 57)	988 (58.8)	285 (32.8)		1273(50.0)	
Mild Steatosis (HU = 57:40)	568 (33.8)	483 (55.6)		1051 (41.2)	
Moderate Steatosis (HU = 40:23)	92 (5.5)	77 (8.9)		169 (6.6)	
Severe Steatosis (HU < 23)	31 (1.8)	24 (2.8)		55 (2.2)	
CACS > 0 (n, %)	768 (45.7)	385 (42.8)		1153 (44.7)	
HbA1c, mmol/mol	35.47 (4.39)	36.11 (7.08)		35.69 (5.49)	
Glucose, mmol/L	5.78 (0.76)	5.94 (1.30)		5.83 (0.98)	
Insulin, mU/L	7.39 (5.12)	7.01 (5.05)		7.26 (5.10)	
HOMA-IR	1.95 (1.49)	1.94 (1.73)		1.95 (1.58)	
TG, mmol/L	1.26 (0.78)	1.28 (0.82)		1.27 (0.79)	
LDL-C, mmol/L	3.76 (0.93)	3.73 (0.93)		3.75 (0.93)	
HDL-C, mmol/L	1.71 (0.50)	1.63 (0.49)		1.68 (0.50)	
CRP, mg/L	2.15 (3.82)	2.28 (5.26)		2.19 (4.38)	
Systolic blood pressure, mmHg	126.32 (15.70)	124.20 (17.30)		125.58 (16.30)	
*Alcohol intake (n, %)*					
Never	70 (4.2)	50 (5.7)		120 (4.7)	
Once a month or less	226 (13.5)	108 (12.2)		334 (13.0)	
2–4 times a month	673 (40.1)	337 (38.2)		1010 (39.4)	
2–3 times a week	599 (35.7)	311 (35.2)		910 (35.5)	
4 times a week or more	110 (6.6)	77 (8.7)		187 (7.3)	

The value attributed to each feature represents the mean value, while in parenthesis is given the SD or the percentage when stated in the first column.

Out of 284 lipids associated with liver attenuation, the plasma levels of 19 lipids were found to also be associated with CACS (Fig. 1). These 19 lipids were increased in relation to both higher liver steatosis and higher CACS. Dihydroceramides and dihydrosphingomyelins were among the groups of lipids with the most pronounced associations. The same lipid that was found to have the strongest association with liver steatosis, N-stearoyl-sphinganine (d18:0/18:0), a dihydroceramide, also had the most significant association with CACS > 0. The lipid 1-palmitoyl-2-oleoyl-GPC (16:0/18:1) had the largest association to CACS.

### Two FFAs show high mediating effect between steatotic liver disease and CAD

**Fig. 3:**
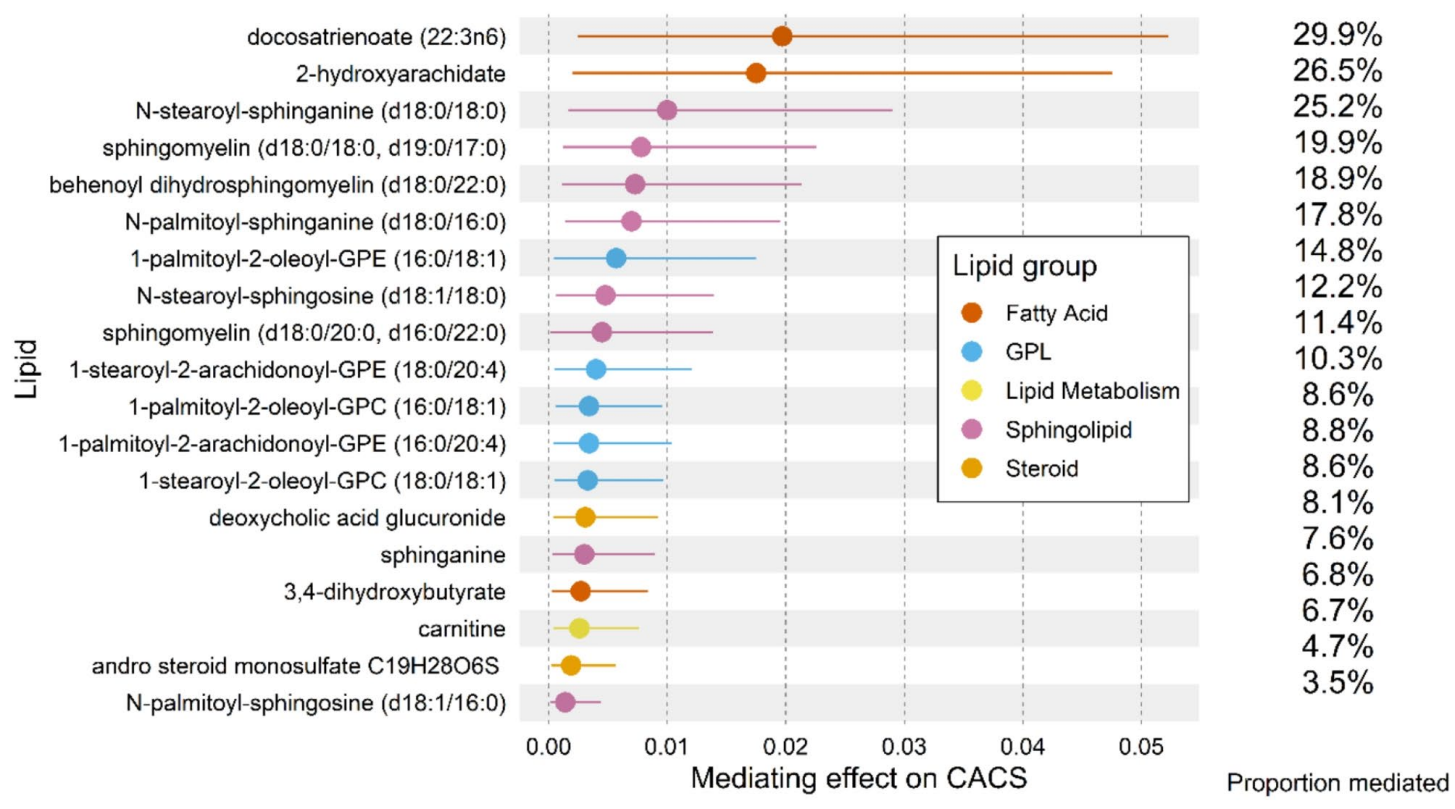
Forest plot of lipids’ ACME with their average proportions mediated adjusted for gender, age, and cohort.

To estimate the total impact of steatotic liver disease on CACS in our cohort, the odds ratio per 1-standard deviation increase in liver steatosis was first calculated (**Supplementary Table 1**), showing that liver fat was independently associated with CACS. We next performed a mediation analysis to investigate the relative importance of these 19 lipids in terms of mediating the association between liver steatosis and CACS. It was found that the two lipids that exhibited the highest ACME between steatotic liver disease and CACS were two FFAs (Fig. 3). The first FFA is docosatrienoate (22:3n6) with an ACME of 0.0197 (95% CI: 0.0025–0.0523). The second is 2-hydroxyarachidate with an ACME of 0.0175 (95% CI: 0.002–0.0475). The correlation matrix of these 19 lipids including TG and LDL-C indicates that these two fatty acids are not closely correlated (**Supplementary Fig. 2**). The groups of sphingolipids and GPLs followed the two FFAs based on their ranked ACME. Sphingolipids and GPLs were the most represented groups, with 8 and 5 lipids belonging to each group respectively, while docosatrienoate (22:3n6) and 2-hydroxyarachidate exhibited at least twice as large ACME as most other lipids.

### The mediating effect of these two FFAs were independent of other risk factors

To examine the robustness of our results, sensitivity analysis was conducted. Only HOMA-IR lowered the ACME and narrowed the confidence interval of most lipids, while the opposite happened in case of BMI, SBP, and CRP (**Supplementary Fig. 3**). Overall, the results were robust against including these covariates. On the other hand, the addition of LDL-C attenuated the mediating role of sphingolipids, while the addition of TG had the same impact on GPLs (**Supplementary Fig. 4**).

## Discussion

In this study, we investigated the mediating role of the plasma lipidome between steatotic liver disease and CAD. We found that more than half of circulating lipids were associated with steatotic liver disease, with 284 out of 458 lipids having altered plasma levels. This provides a comprehensive assessment of plasma lipid alterations across various lipid groups in a large population in comparison to previous research^17–20^. Among these 284 lipids, 19 were found to have increased plasma levels in both steatotic liver disease and CACS > 0. Of these 19 lipids, we identified two free fatty acids that independently mediated the effects of steatotic liver disease on CAD.

The main finding of this article is that two free fatty acids were identified as top mediators between steatotic liver disease and CAD. Research on both docosatrienoate (22:3n6) and 2-hydroxyarachidate is limited. Regarding docosatrienoate (22:3n6), a previous study demonstrated that it exhibits the highest fold change among metabolites measured in liver cells of humanized mice with liver steatosis in comparison to mice in the control group^21^. In general, N-6 PUFAs are implicated in promoting inflammation as the precursors of pro-inflammatory molecules^22^. Another study in mice showed that exposure to an organic pollutant increased the amount of docosatrienoate (22:3n6) in the liver, while increasing inflammation and exacerbating atherosclerosis^23^. In relation to 2-hydroxyarachidate, the only existing information in connection to steatotic liver disease is, to our knowledge, an observed elevation in the plasma of individuals with severe insulin-resistant diabetes^24^.

The next two groups of lipids according to the ranked ACMEs were sphingolipids and GPLs. When adjusting for LDL-C the role of sphingolipids were attenuated, whereas adjusting for plasma TG attenuated the role of GPLs (**Supplementary Fig. 4).** Since both LDL-particles and triglyceride-rich lipoproteins cause CAD, sphingolipids and GPLs do not appear to serve as independent risk factors for CAD. Consequently, the mediating effect of these two lipid groups may be explained by the role of constituents of atherogenic lipoproteins. However, the inclusion of LDL-C and TG did not affect the role of docosatrienoate (22:3n6) and 2-hydroxyarachidate. This suggests that they could mediate increased CAD risk independently from their role as constituents of lipoproteins.

A major limitation of this study is its observational nature, which precludes the establishment of causality; hence, the mediation results should not be interpreted as definite evidence of a causal relationship from steatotic liver disease via the mediators to CAD. Another limitation of the study is that the complexity of the relationships between liver steatosis, lipids, and CACS makes it challenging to precisely quantify the mediating role of these lipids since some of these lipids may further aggravate steatotic liver disease, for example, through inflammatory processes. Moreover, calculating the proportion mediated is based on the estimations of the ACME and total effect and their standard errors. Therefore, the mediation analysis primarily aids in identifying mediating lipids and ranking their importance, rather than providing precise mediation estimates. Another limitation may be that CT was used for estimating liver steatosis. MRI serves as the standard noninvasive method for assessing liver steatosis, non-contrast CT is a reliable method for quantifying liver fat given its strong linear correlation with MRI-derived values^25^, however CT suffers from lower sensitivity and hence there is a risk that some individual’s liver fat was not detected. The major cause of liver steatosis in this cohort is likely to be metabolic-dysfunction. However, while alcohol consumption could be adjusted for in our analyses, the potential influence of rarer causes such as viral and autoimmune liver diseases cannot be completely ruled out. Future studies are needed to establish whether the findings reported in this study are reflected in individuals with confirmed MASLD where other causes are ruled out. Furthermore, it is difficult to exclude the possibility that the increase in plasma levels of these lipids precedes liver steatosis using observational data, although the mediating effects of docosatrienoate (22:3n6) and 2-hydroxyarachidate are not affected by BMI and HOMA-IR, which are known risk factors for liver steatosis. Nevertheless, their significant associations with liver steatosis and atherosclerosis underscore their importance as common denominators of these conditions. Finally, the absence of a validation cohort with definitive cause of SLD impedes our ability to validate our findings and establish their robustness. Future research in this area may strengthen the validity of the conclusions drawn.

## Conclusion

Our findings suggest that steatotic liver disease is associated with changes in the plasma lipidome that may have implications for coronary artery disease. The lipidomic profiling revealed that a substantial proportion (62%) of the 458 lipids examined were associated with liver steatosis. Importantly, two specific fatty acids, namely docosatrienoate (22:3n6) and 2-hydroxyarachidate, were identified as mediators between steatotic liver disease and coronary artery calcification, distinct from other known atherosclerotic risk factors. Further research is needed to elucidate any potential causal pathways and confirm these associations in longitudinal studies.

## Electronic supplementary material

Below is the link to the electronic supplementary material.

Supplementary Material 1

## Data Availability

Data may be shared upon request to the corresponding author.

## Abbreviations

SLD: Steatotic liver disease
CACS: Coronary artery calcium score
IGT-study: Microbiota, development of type 2 diabetes and cardiovascular disease study
SCAPIS: Swedish CArdioPulmonary bioImage Study
CT: Computed tomography
HU: Hounsfield units
CRP: C-reactive protein
HbA1c: Hemoglobin A1c
TG: Triglycerides
SBP: Systolic blood pressure
HOMA-IR: Homeostatic model assessment for insulin resistance
GPL: Glycerophospholipid
