# Supplementary material for: The impact of steatotic liver disease on coronary artery disease through changes in the plasma lipidome
Source: Sci Rep. 2024 Sep 27;14:22307. doi: 10.1038/s41598-024-73406-8 (PMC11436983; doi:10.1038/s41598-024-73406-8)
Supplement: Supplementary file 1 — Supplementary Material 1 [file 41598_2024_73406_MOESM1_ESM.pdf]

Supplementary Material

Additional covariate	Odds ratio (CI 95%)	P-value
None	1.24 (1.13-1.36)	2.39e-06
BMI	1.16 (1.04-1.29)	0.00811
CRP	1.21 (1.11-1.33)	3.5e-05
HOMA-IR	1.21 (1.09-1.34)	0.000276
SBP	1.19 (1.08-1.3)	0.000242
LDL-C	1.23 (1.12-1.34)	7.36e-06
TG	1.2 (1.1-1.32)	0.000159

Supplementary Table 1. Relationship between liver steatosis and CACS. Odds ratio for presence of CAD (CAC score > 0) is expressed per one standard deviation higher liver fat. All models are adjusted for age, sex and study cohort. Further adjustments (i.e. one at a time in addition to age, sex and study cohort) for BMI, CRP, HOMA-IR, systolic BP, LDL-C and TG are also shown

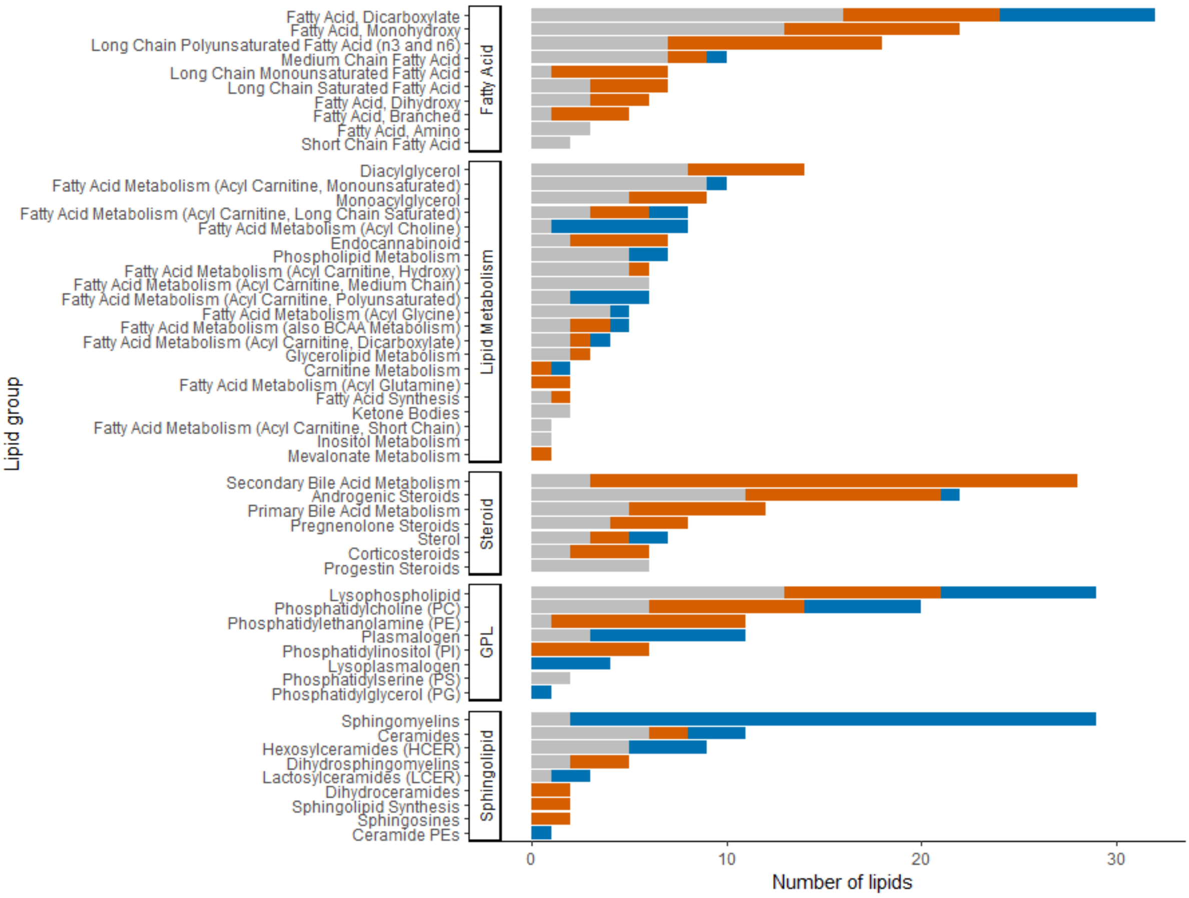

Supplementary Figure 1. Lipidomic profile of liver steatosis adjusted for age, gender, cohort, alcohol consumption, and BMI.

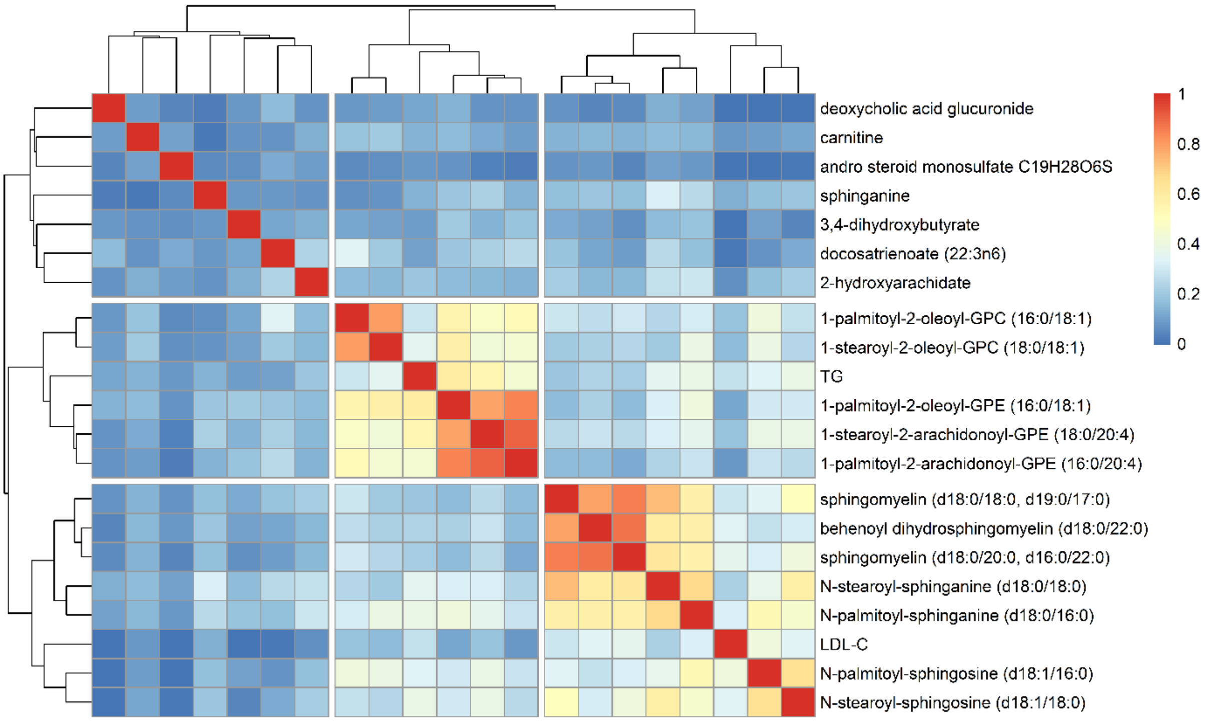

Supplementary Figure 2. Correlation matrix heatmap of mediating lipids including LDL-C and TG. Color shades closer to red represent high correlation between adjacent lipids, while shades of blue represent low correlation.

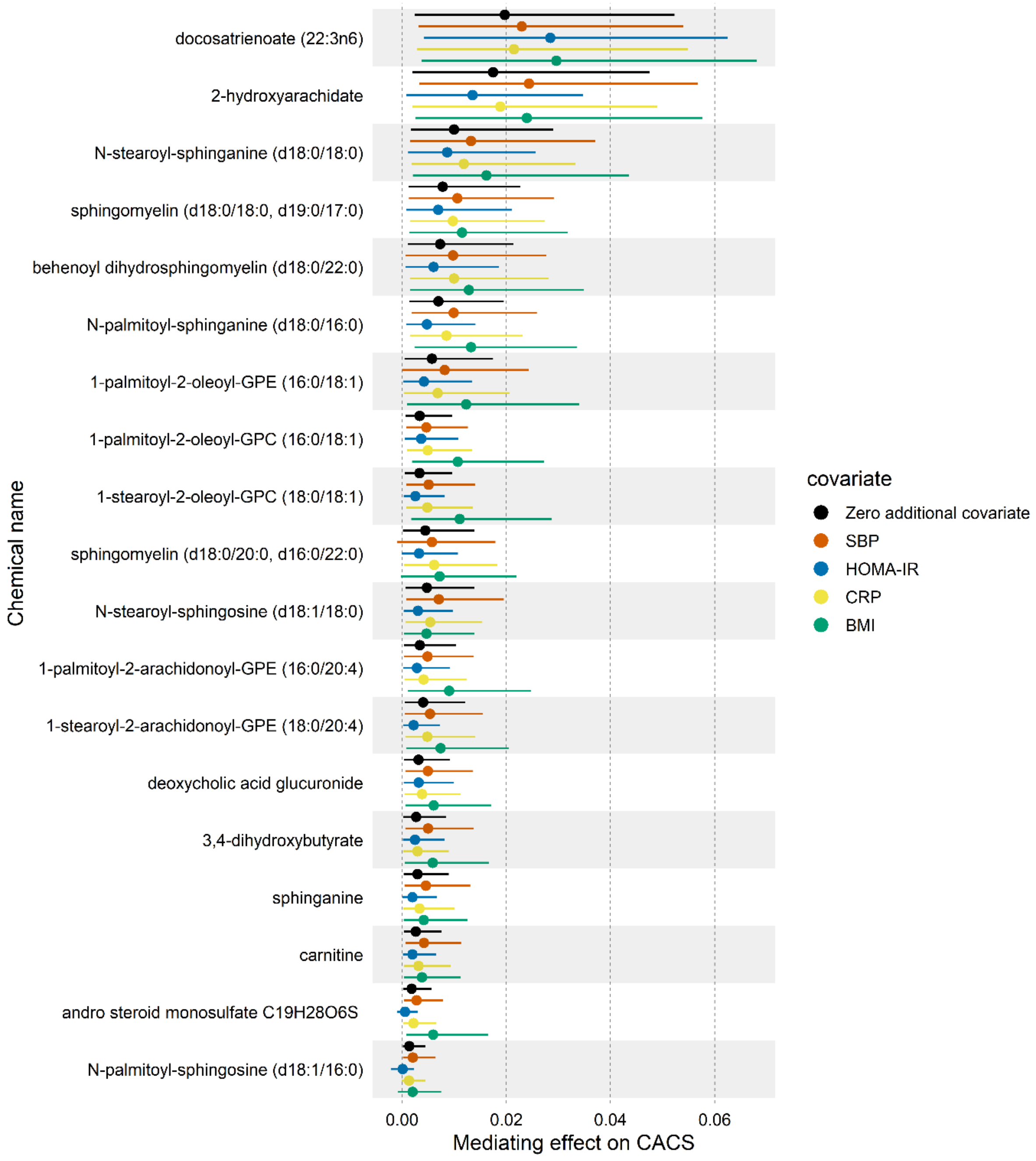

Supplementary Figure 3. Sensitivity analysis. Forest plots of lipids' ACME in model with gender, age, and cohort as covariates plus the respective addition of SBP, HOMA-IR, CRP, and BMI.

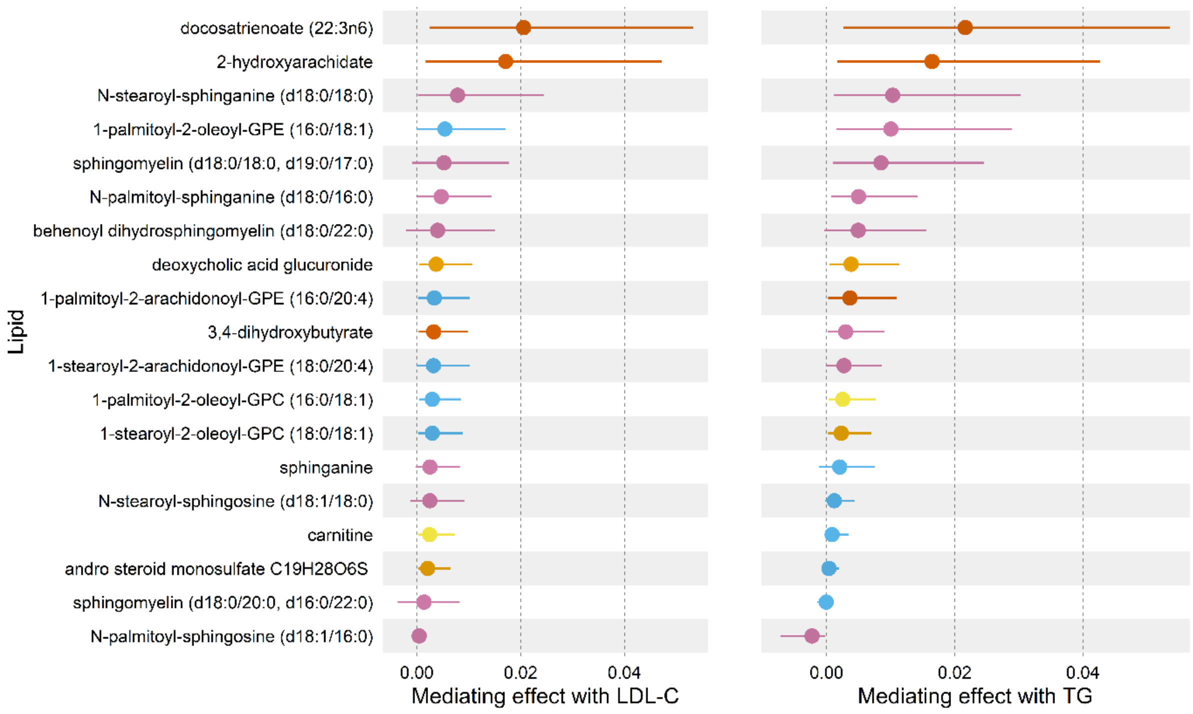

Supplementary Figure 4. Sensitivity analysis. Forest plots of lipids' ACME in model with gender, age, and cohort as covariates plus the respective addition of LDL-C and TG. (Colors: red for fatty acids, pink for sphingolipids, sky-blue for GPLs, yellow for lipid metabolites, and orange for steroids.)
